# Spanish Outbreak Isolates Bridge Phylogenies of European and American *Bacillus anthracis*

**DOI:** 10.3390/microorganisms11040889

**Published:** 2023-03-29

**Authors:** Olga Bassy, Markus Antwerpen, María Victoria Ortega-García, María Jesús Ortega-Sánchez, José Antonio Bouzada, Juan Carlos Cabria-Ramos, Gregor Grass

**Affiliations:** 1 Chemical, Biological, Radiological and Nuclear (CBRN) Defence Systems Department, Campus La Marañosa, Instituto Nacional de Técnica Aeroespacial “Esteban Terradas” (INTA), 28330 San Martín de la Vega, Madrid, Spain; 2 Bundeswehr Institute of Microbiology (IMB), 80937 Munich, Germany; 3 Laboratorio Central de Sanidad Animal (LCSA), Ministerio de Agricultura Pesca y Alimentación (MAPA), 18320 Santa Fe, Granada, Spain; 4 Laboratorio Central de Veterinaria, Ministerio de Agricultura Pesca y Alimentación (MAPA), 28110 Algete, Madrid, Spain

**Keywords:** anthrax, *Bacillus anthracis*, Spain, genotyping, genome sequencing, phylogeny

## Abstract

The geographical origin of a major present-day phylogenetic group (A branch WNA; A.Br.WNA) of American *Bacillus anthracis* is controversial. One hypothesis postulated that the anthrax pathogen reached North America via a then-existing land bridge from northeastern Asia thousands of years ago. A competing hypothesis suggested that *B. anthracis* was introduced to America a couple of hundred years ago, related to European colonization. The latter view is strongly supported by genomic analysis of a group of French *B. anthracis* isolates that are phylogenetically closely related to the North American strains of the A branch A.Br.WNA clade. In addition, three West African strains also belong to this relationship group. Recently, we have added a Spanish strain to these close relatives of the WNA lineage of American *B. anthracis*. Nevertheless, the diversity of Spanish *B. anthracis* remains largely unexplored, and phylogenetic links to European or American relatives are not well resolved. Here, we genome sequenced and characterized 29 new *B. anthracis* isolates (yielding 18 unique genotypes) from outbreaks in west central and central Spain in 2021. Applying comparative chromosomal analysis, we placed the chromosomes of these isolates within the established phylogeny of the A.Br.008/009 (A.Br.TEA) canonical SNP group. From this analysis, a new sub-clade, named A.Br.11/ESPc, emerged that constitutes a sister group of American A.Br.WNA.

## 1. Introduction

Anthrax is a zoonotic disease mostly affecting herbivorous mammals that typically become infected by soil-borne endospores of the etiological agent, *Bacillus anthracis*. Humans acquire the disease mostly by slaughtering and eating undercooked meat from infected animals or by the handling of contaminated animal products such as hides or wool [1]. In the past, anthrax has been an almost worldwide disease (except for polar regions and certain islands) but coordinated control measures have diminished anthrax prevalence in most of Europe, North America and parts of Asia and Australia [1].

In Spain, anthrax is also a rare disease, with eight confirmed cases of anthrax in humans from 2002 to 2020, most of them cutaneous anthrax (ECDC, Annual Epidemiological Reports; https://www.ecdc.europa.eu/en/publications-data/monitoring/all-annual-epidemiological-reports (accessed on 25 March 2023)). Historically, anthrax was an endemic disease in Spain up to the 20th century. Since the 1970s, the number of cases has drastically decreased thanks to vaccination and antibiotic treatment. However, human cases continue to be reported, including in the regions affected by the two outbreaks referred to in this study. Anthrax is largely an occupational disease [1]. The source of these human cases is typically animals and the absence of registered cases in animals (due to non-specific clinical signs) would thus not exclude the absence of the disease. Indeed, anthrax would appear sporadically and very localized in these regions, as has been observed in the past. The last anthrax outbreak officially registered in animals in Spain was in 2004. Surprisingly, 2021 has been an active anthrax year with a variety of human and animal cases in the west central and central Spanish regions (Extremadura and Castile-La Mancha, respectively).

Little is known about the previous or extant diversity of *B. anthracis* in Spain. A single isolate was genome sequenced in 2016 as part of a global phylogeny study [2]. Later, the genomes of two additional strains were analyzed and found to belong to a major clade of *B. anthracis*, the so-called A Branch 008/009 (A.Br.008/009) canonical SNP group [3], also known as A.Br.TransEurasian [4]. Notably, these isolates are grouped close or very close to strains from southwestern Europe and from North America [3]. The single, nearly exclusively North American lineage A.Br.WNA (Western North American; also known as A.Br.009) is a bit of a conundrum as two competing hypotheses on its origin have been formulated [5,6]. Initially, it was suggested that the anthrax pathogen reached the North American continent during the late Pleistocene epoch via the then-existing Beringia land bridge between Alaska and Siberia [5]. Alternatively, *B. anthracis* could have been introduced much later, along with European exploration, conquest and settlement during the Age of Discovery about 500 years ago [6]. Supporting the latter hypothesis, a group of French *B. anthracis* isolates of the A.Br.011 canonical SNP group exhibits a close phylogenetic relationship to typical North American strains of the A.Br.WNA clade, which is a subclade of A.Br.011 [2,6]. Conversely, eastern Asian strains belong to other canSNP groups, typically A.Br.001/002 (Sterne) or A.Br.001 (Ames) [2,7].

In this study, we genome-sequenced and analyzed additional *B. anthracis* strains associated with recent outbreak events in Spain. Phylogenetic placement of these isolates near the diverging node of the American A.Br.WNA clade supports the modern-age introduction of founding ancestors of this group from somewhere in southwest Europe, possibly Spain.

## 2. Materials and Methods

### 2.1. Growth of B. anthracis and Extraction of DNA from Inactivated Culture Material

All live *B. anthracis* strains were handled in a biosafety level 3 (BSL-3) laboratory at *Laboratorio Central de Sanidad Animal* (LCSA), Santa Fe (Granada), Spain. Cultures of *B. anthracis* (Table 1) were grown on blood agar or PLET agar and then inactivated by heating before further use [1]. DNA was isolated using a QIAmp DNA Mini kit (Qiagen, Hilden, Germany) as described for Gram-positive bacteria. DNA concentrations were quantified at the *Instituto Tecnológico Agrario de Castilla y León* (ITACyL) using the Qubit^®^ Fluorometer 4.0 (Invitrogen, ThermoFisher Scientific, Waltham, MA, USA) and the Qubit dsDNA BR Assay Kit (Invitrogen). DNA preparations were stored at −20 °C until further use.

### 2.2. Whole Genome Sequencing

Whole genome sequencing was performed using the Illumina MiSeq platform with 2 × 300 bp v3-chemistry at ITACyL or IMB as follows. A compatible library using a Nextera XT DNA Library Preparation kit (Illumina, San Diego, CA, USA) or an Illumina DNA Prep kit (Illumina) was prepared by adjusting the concentrations to 4 nM, so that each library was calibrated at an equimolar concentration in a single pool of samples. The libraries were normalized and mixed into a pool, denatured with 0.2 N NaOH and loaded into the sequencer with PhiX Sequencing Control v3 (Illumina) at different percentages depending on the reaction. Reads that did not meet the length and quality criteria were filtered using Prinseq v0.20.4 [8]. Kraken v2.0.9-beta software [9] was used to classify the sequences taxonomically as additional quality control. High-quality paired-end reads (Q ≥ 30) were assembled de novo using an in-house script based on the SPAdes (version 3.15.2) assembler [10] to create draft genomes, and Pilon (version 1.22) [11] was used for correcting SNPs or closing small gaps and INDELs. Assembly statistics (number of contigs, length, N50, depth, etc.) were calculated with QUAST v5.0.2 [12]. The obtained scaffolds were manually checked for contaminant reads and annotated with Prokka v1.14.6 [13]. All data generated or analyzed during this study are included in this article and its supplementary information files. Genome sequence data are publicly available in the NCBI Sequence Read Archive (SRA) repository (Bioproject PRJNA930337).

### 2.3. Analysis of Whole Genome Sequencing Data–SNP Calling

For rapid core chromosome multiple alignment, the Parsnp tool from the Harvest Suite was used [14]. For this, a chromosome dataset, representing genomes from public databases (Table S2) and the newly sequenced strains of *B. anthracis*, were aligned against the chromosome of *B. anthracis* ‘*Ames ancestor*’ (accession number NC_007530) as a phylogenetic outgroup using Parsnp (parameters -c -e -u -C 1000). To export the identified SNP positions, HarvestTools (version 1.2) from the same software suite was used to create a vcf (Variant Calling File) listing all SNP positions. In order to enhance data quality, chromosome regions with closely adjacent SNPs (<10 bp distance), as well as positions harboring undefined nucleotides (“N”), were removed. This curated vcf file was used as an input for HarvestTools to compile a multi-FASTA file out of the chromosome dataset comprising the concatenated SNPs as a multiple-sequence alignment. This concatenated sequence information was used to calculate a maximum parsimony tree in MEGA X [15,16]. The SNPs identified within the analyzed *B. anthracis* chromosomes are compiled in Table S3. In addition, a minimum spanning tree was computed using an in-house script based on R [17] using the vcf SNP file (in binary format) as an input. Trees were manually edited (using Powerpoint, Microsoft, Redmond, WA, USA) for style and labeling.

### 2.4. Interrogation of SNPs via PCR by Relative Ct Value Analysis (Delayed Mismatch Amplification Assay)

Delayed mismatch amplification PCR assays (DMAA) [3] were used to experimentally determine the distribution of selected SNPs in additional *B. anthracis* DNA. This probe-free, real-time qPCR assay interrogates the character state of SNPs (derived vs. ancestral) in two separate PCR reactions containing matching and mismatching forward primer oligonucleotide preferentially favoring amplification of one of the two alleles. Analysis of PCR runs is accomplished by simple relative subtraction of numerical PCR threshold values (Ct values) [3]. DMAA SNP primer oligonucleotides for new SNPs were designed surrounding the SNP positions using the Geneious Prime software (Dotmatics, Bishops Stortford, UK) with the *B. anthracis* Ames ‘Ancestor’ chromosome (accession # NC_007530) as an (ancestral) reference. DMAA-SNP primer sequences for real-time PCR assays are listed in Table S4. Each primer pair was used in a 20 μL single-plex reaction. For this, 1 μM of each primer pair, and approximately 20 ng of the template DNA were added to 1 × LightCycler Fast Start DNA Master SYBR Green I Master mix (Roche, Mannheim, Germany). Amplification and analysis of Ct values were carried out on a LightCycler 2.0 instrument (Roche, Mannheim, Germany) as described in [18], without melting curve analysis.

## 3. Results

### 3.1. Recent Outbreaks of B. anthracis in West Central and Central Spain

**Figure 1:**
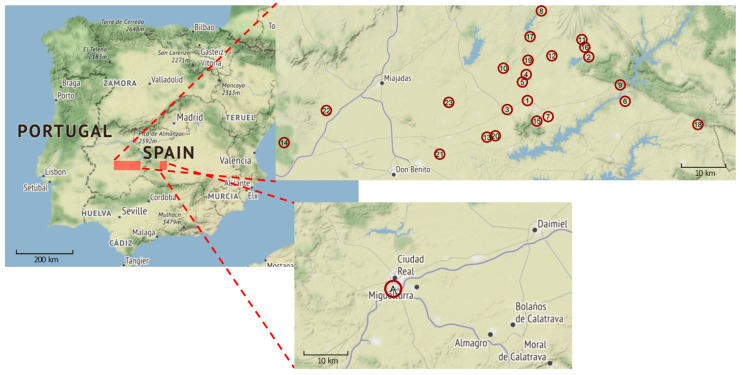
Outbreaks of *B. anthracis* in west central and central Spain from August 19 to 14 December 2021. A map of Spain is shown (**center-left image**) with close-ups of the two outbreak regions. The first outbreak took place near the city of Ciudad Real (**lower-right image**), and the second covered several villages in the Extremadura region (**upper-right image**). Red circles indicate the location of the affected farms, numbered chronologically by the start date of the outbreaks (Extremadura map) or alphabetically (Ciudad Real map). For details of these outbreaks, see Table S1. Data were extracted from the Spanish reports sent to the OIE (https://www.woah.org/). Map tiles by Stamen Design, under CC BY 3.0 license. Data from OpenStreetMap, under ODbL.

In 2021, there were two independent anthrax outbreaks in Spain (Figure 1; Table 1 and Table S1). The first outbreak took place in the outskirts of Ciudad Real, from August 19 to September 3. Only a single farm suffered 25 infected cattle. In this outbreak, the source of infection was the pasture because only animals put to grazing there were affected. Animals from the same farm staying in the feedlot did not contract the disease. This area is usually covered by the flow of the Guadiana River but had been uncovered due to drought. Likely, this dried-up riverbed represented the origin of the pathogen. As soon as the animals were confined, new cases stopped appearing, and the outbreak remained restricted to this single farm (farm A in Figure 1; Table 1 and Table S2). Environmental sampling was not carried out during or after this outbreak.

The second outbreak (Figure 1; Table 1 and Table S1) began in Navalvillar de Pela (Badajoz) on August 25 and spread through several towns in Badajoz and Cáceres, with 23 farms affected and a total of 129 infected animals (cattle, horses or pigs and one wild boar) and two human cases. The outbreak was finally brought under control on December 14 but its origin remains unknown. Environmental sampling was carried out yielding 67 environmental samples (soil, straw, hay, water and feed) and 30 samples from insect traps. *B. anthracis* was isolated from a soil sample related to farm 19 in Logrosán. The role of insects is suspected as they might have helped to disperse the disease, as DNA of *B. anthracis* was detected in an insect sample from farm 21 in Villanueva de la Serena. Before this outbreak, too, riverbeds had dried up due to drought in 2021, possibly exposing previous anthrax foci. Geographically, farm 8 was the northernmost premise with infected animals, farm 21 the southernmost, farm 18 the easternmost and farm 14 the westernmost. Farm 13 (in Acedera) is located 35 km away from farm 9 (in Talarrubias). Farm 14 (in Mérida) is 103 km away from farm 18 (in Garbayuela), and the distance is 44 km from farm 21 (in Villanueva de la Serena) to farm 8 (in Logrosán). Thus, the outbreak area in the Extremadura region was about 4000 km^2^.

### 3.2. A B. anthracis Lineage Comprising Strains from Spain Constitutes a Sister Clade to a Clade of North American Strains

**Figure 2:**
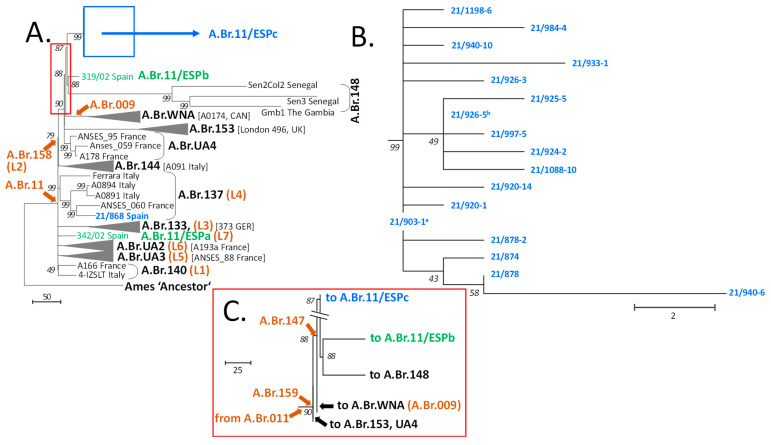
Rooted phylogenetic tree of Spanish *B. anthracis* and representatives of the A.Br.011/009 canonical single-nucleotide polymorphisms (canSNP) clade derived from chromosomal SNP analysis. A total of 1817 chromosomal SNPs were used to construct a maximum parsimony tree (bootstrap confidence values were generated from 500 permutations). (**A**) Overview tree to illustrate the general positions of Spanish strains within the A.Br.011/009 phylogeny. (**B**) Close-up of the diversity of new branch A.Br11/ESPc. (**C**) Close-up of branching after canSNP A.Br.159. To indicate the positions of additional branches, these are drawn as condensed down to a single representative (grey wedges with strain names in brackets). Branch names or relevant canSNP positions are set in black or brown bold characters, respectively. Previously identified Spanish strains and branches are colored in green and new strains and clades in blue. All other relevant isolate names and countries of origin are indicated at branch termini. The red and blue boxes in panel A indicate the sections shown in detail in panels B and C. The tree is rooted to the *B. anthracis* reference strain Ames ‘Ancestor’, which belongs to the A.Br.Ames clade. ^a^ identical chromosomal sequence to isolates 21/890-8; 21/890-9; 21/903-6; 21/905-13; 21/918-3; 21/926-9; 21/926-22; 21/940-19 and 21/955-9; ^b^ identical chromosomal sequence to isolates 21/891-2 and 21/1088-3.

From the recent *B. anthracis* outbreaks in Spain, 29 isolates were genome sequenced. Initial mapping of the resulting 29 chromosome sequences against the Ames ‘Ancestor’ reference revealed that there were only 18 unique genotypes (Table 1). All belonged to the canSNP group A.Br.011/009. Duplicate sequences (11) were omitted from further analysis and the 18 new Spanish *B. anthracis* chromosomes were compared to 19 previously sequenced chromosomes from canSNP group A.Br.011/009 [3] and the reference as root (Table S2). These 19 strains were selected to represent members of previously defined A branches (A.Br.) including close individual relatives of Spanish *B. anthracis*. From this, a total of 1817 chromosomal SNP positions were identified (Table S3). These SNP data were used to calculate a maximum parsimony tree (Figure 2A–C).

In this tree, Spanish *B. anthracis* isolates were grouped within four different clades, all positioned distal to canSNP A.Br.011 (i.e., possess a derived character for this SNP) (Figure 2A). Three of these clades exclusively comprised Spanish strains and were named A.Br.11/ESPa (L7) to A.Br.11/ESPc. Clades A.Br.11/ESPa (L7) and A.Br.11/ESPb both harbored a single member only. The vast majority of new Spanish genomic sequences (17 out of 18) belonged to newly established clade A.Br.11/ESPc and appeared to be very closely related to one another (Figure 2B). Of note, we observed a shallow polytomy at the base of this clade with two sub-branches and eight single isolates.

The fourth clade containing a newly isolated Spanish *B. anthracis* was clade A.Br.0137 (L4). With the addition of a new Iberian strain, this group then comprised southwestern European isolates from Italy, France and Spain (Figure 2A).

Figure 2C highlights the branching region between A.Br.11/ESPc and A.Br.WNA (A.Br.009) comprising the branches with derived character states for canSNPs A.Br.159 and A.Br.147. In this representation, clade A.Br.WNA branched off first after passing canSNP A.Br.147, from a lineage leading to both clade A.Br.11/ESPc and to clades A.Br.11/ESPb along with A.Br.148. All these shared the derived character state for canSNP A.Br.147. This analysis suggests that two groups of Spanish *B. anthracis* isolates (clades A.Br.11/ESPb and A.Br.11/ESPc) might represent the closest extant relatives of the American A.Br.WNA clade.

### 3.3. Relationships among Spanish B. anthracis Strains and with Their Relatives from Europe, Africa and America

**Figure 3:**
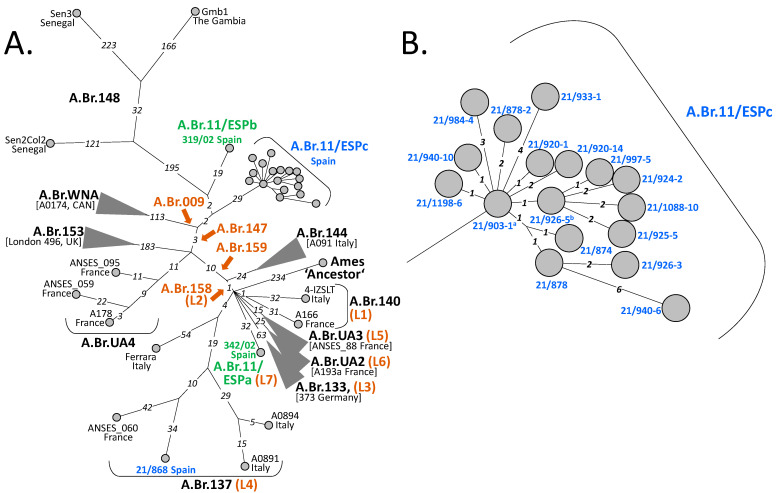
SNP distances between Spanish isolates of *B. anthracis* and their relatives of the A.Br.011/009 lineage. Minimum spanning trees of Spanish *B. anthracis* isolates and representative individual relatives with (condensed) clades (**A**) or a close-up of members of clade A.Br.11/ESPc (**B**) are depicted. Shown are numerical SNP distances between chromosomes of isolates from Figure 2. Color-coding of branch names, condensed branches, strains or relevant canSNP positions is the same as in Figure 2. The tree is rooted to the *B. anthracis* reference strain Ames ‘Ancestor’ (from the A.Br.Ames clade). ^a^ identical chromosomal sequence to isolates 21/890-8; 21/890-9; 21/903-6; 21/905-13; 21/918-3; 21/926-9; 21/926-22; 21/940-19; 21/955-9; ^b^ identical chromosomal sequence to isolates 21/891-2; 21/1088-3.

Additional analysis of the A.Br.008/009 canSNP clade focusing on SNP distributions within chromosomes of Spanish *B. anthracis* isolates revealed additional details (Figure 3). Evidently, in contrast to other branches harboring Spanish strains (Figure 3A), the diversity within the polytomic A.Br.11/ESPc clade was quite shallow (Figure 3B). The maximum distance within this group was only 12 SNPs. Most direct distances between any two isolates were ≤6 SNPs. In relation to the outbreak events from which these isolates were obtained (Table S1), the following correlations can be established. The SNP distances between affected farms are as follows. Farm 9 in Talarrubias (Figure 1 and Table S1) is the location from where strain 21/903-1 was isolated. This one is the most basal genome of clade A.Br.11/ESPc (Figure 2 and Figure 3). From farm 13 (in Acedera), the strain featuring the highest number of SNPs (isolate 21/940-6 featuring 8 SNPs) was retrieved. These 8 SNPs represented the greatest phylogenetic distance to the base of this A.Br.11/ESPc clade. In these two farms, the outbreaks took place between September 8 (start date of the outbreak in farm 9) and October 10 (end date of the outbreak in farm 13) (Table S1).

Regarding chromosomal sequence-identical isolates, isolate 21/903-1 was identical to isolates 21/890-8, 21/890-9, 21/903-6, 21/905-13, 21/918-3, 21/926-9, 21/926-22, 21/940-19 and 21/955-9; isolate 21/926-5 was identical to isolates 21/891-2 and 21/1088-3 (Figure 2 and Figure 3). Of note, both groups were only separated by a single SNP.

There are 23 km between farm 9 (isolate 21/903-1; basal genotype) and farm 4 (isolates 21/890-9 and 21/903-6). Farms 4 and 5 (21/890-8, and 21/905-13, respectively) are next to each other. The distance is the same (about 20 km) between farms 9 and 15 (isolate 21/926-9) and between farms 9 and 18 (isolate 21/955-9), respectively. However, the distance between farm 14 (isolate 21/926-22) and farm 9 (isolate 21/903-1; basal genotype) is 83 km. For the other group of sequence-identical isolates (Figure 2 and Figure 3), the distance, e.g., from farm 7 (isolate 21/891-2) to farm 21 (isolate 21/1088-3) is 28 km but between farms 7 and 18 (isolate 21/926-5) the distance is 37 km. Thus, there is a wide geographical spread of identical genotypes among places with unique singletons. Therefore, we cannot draw a clear spatial correlation between these strains. Notably, there were farms from which two to three different genotypes were collected. Farms 5, 17 and 18 yielded two genotypes each (21/903-1 and 21/920-14 or 21/926-3 and 21/933-1, or 21/926-5 and 21/903-1, respectively) featuring two, eight and one SNPs. Farm 13 produced three genotypes (21/924-2, 21/925-5, 21/940-6) separated by up to 11 SNPs (Figure 3B and Table S1).

Finally, the distance from the first, unrelated outbreak, i.e., farm A (in Ciudad Real), to farm 18 (in Garbayuela) of the second outbreak is only 93 km. In other words, the geographical distances within the second outbreak, giving rise to strains in clade A.Br.11/ESPc, were partly almost as great as the distance to the non-related outbreak at farm A featuring strain 21/868 from clade A.Br.137 (L4).

The hypothetical base of the lineage leading to branch A.Br.11/ESPb (strain 319/02 from Spain) was only two SNPs distant from the base of clade A.Br.11/ESPc (Figure 3A). Strain 319/02 had been isolated in Guadalajara province approx. 260 km away from Talarrubias (Badajoz) and 19 years earlier than strain 21/903-1. Clearly, lineages A.Br.11/ESPb and A.Br.11/ESPc share a common, probably relatively recent, ancestor. Notably, the base of these two purely Spanish lineages (A.Br.11/ESPb and A.Br.11/ESPc) also gave rise to the West African lineage A.Br.148 (Figure 3A).

In contrast, Spanish strain 21/868, isolated from a bovine near Ciudad Real at the beginning of September 2021, was nested within strains from France and Italy at considerable individual distances. Even the closest relative, strain ANSES_060 from dept. Tarn in France, isolated ca. 1900, was 76 SNPs distant. The largest distance showed strain Ferrara from Italy with 117 SNPs (Figure 3A). Lastly, Spanish strain 342/02 branched off directly from a polytomy at the base of the A.Br.009/011 clade, giving rise to a lineage, A.Br.11/ESPa (L7), with strain 342/02 being the single member harboring 32 autapomorphic SNP positions (Figure 3A).

Currently, we know that clade A.Br.11/ESPc, detected in Extremadura forming a single clonal cluster, was distributed over an area of at least 4000 km^2^. On the other hand, the genotype detected in Ciudad Real, which is 112 SNPs distant from strain 21/903-1, is more similar to other isolates of non-Spanish but French or Italian origin. For the time being, it seems there is widespread contamination in west central and central Spain with similar but clearly distinct genotypes of *B. anthracis* without any strong spatial correlation. Thus, the Spanish *B. anthracis* isolates available for this study all clustered within only one of the major canSNP groups of this organism (A.Br.009/011). Within this clade, their distribution was not scattered across this phylogeny but instead, three of the four lineages featuring Spanish isolates comprised Spanish isolates only.

### 3.4. Confirmation of Results by SNP Discriminating PCR Assays (DMAA)

In an effort to genotype additional Spanish isolates concerning their attribution to lineages described herein, especially the new clade A.Br.11/ESPc, three new DMAA-PCRs were tested as a proof of principle. The scored character state of sub-clade SNP A.Br.11/ESPc for one *B. anthracis* isolate from the Extremadura outbreak (21/874) was derived (as expected from genome sequencing). In contrast, DNA of a *B. anthracis* isolate (VV-E-815) from a Spanish anthrax focus (not part of this study) was ancestral, as were other Spanish clinical isolates (319/02 and 342/02) belonging to their unique lineages (A.Br.11/ESPb and A.Br.11/ESPa (L7), respectively). Thus, in case of new outbreaks in the Extremadura region of Spain, these facile PCR tests may be employed for rapid initial genotyping.

## 4. Discussion

*B. anthracis* is a very monomorphic species with little horizontal gene transfer and few genomic rearrangements between different isolates [19]. This makes the analysis of genomic SNPs an ideal global genotyping tool providing high resolution for phylogenetic placements of new isolates [6]. Notably, for the phylogeny of the major canSNP lineage A.Br.011/009 of *B. anthracis*, there is a polytomy at the base of this clade [6]. Radiating from this remarkable node (i.e., the most recent common ancestor, MRCA), seven branches [20] spread out (Figure 3A). From these, one lineage (A.Br.158; L2) eventually leads to clade A.Br.WNA. Vergnaud et al. (2016) have suggested that the North American A.Br.WNA clade [5] is a recent lineage from a post-Columbian introduction from Europe [6]. The authors argued that the shortest terminal branch from this polytomy node (the MRCA) is quite short (17 SNPs). Thus, the higher number of SNPs observed in A.Br.WNA genomes acquired within the same time period (since divergence) must be due to a higher mutation rate, vis-á-vis, a higher number of generations since divergence [6]. The same authors offer Western Europe (specifically France) as the likely region of origin of WNA. They favored a post-Columbian introduction to the New World and hypothesized the temporal placement of the A.Br.011/009 polytomy’s MRCA. In the current model, now three countries, France, Spain or Italy are favored as the places of this origin. Possibly, military operations have inadvertently fueled the spread of the MRCA’s descendants radiating from an initial outbreak. Thus, the birth of the A.Br.011/009 polytomy and its founder lineages is thought to be associated with these human activities between the three countries [20].

The exploration of the Americas by the Spanish conquerors was the largest and the longest if compared to that of other overseas nations in that continent [21]. From California peninsula to Louisiana in the north until Tierra de Fuego in the south [21], and from the 1500s to the 1800s [22]. Half of American land has been part of the Spanish Empire at some time [21] with sprawling metropolitan areas and major roads connecting the population centers. Notable is the scarcity of domestic animal species in the New World at the arrival of the Spanish conquerors. Only five animal species were domesticated in (parts of) the pre-Columbian Americas: turkey, llama/alpaca, guinea pig, musk duck and dog [23]. This lack of indigenous animal resources, among other reasons, could explain the introduction of anthrax by unaware Spanish conquerors via their domesticated cattle and horses, among other European domestic animals or their by-products.

While we are mindful of the historical events described above [20] causing phylogenetic ambiguities, we propose Spain as the region with a high likelihood of giving birth to the MRCA of European A.Br.147 strains evolving into the American A.Br.WNA clade. Our new data are in agreement with a post-Columbian introduction of *B. anthracis* from Europe to America. In our redefined hypothesis, the focus shifts away from France as the origin country. This hypothesis is supported by the phylogenetic analysis of SNP distribution. However, without taking into account the historical interactions between France, Spain and Italy [20], in principle, extant Spanish *B. anthracis* strains could have secondarily been introduced into the Iberian Peninsula from other southwestern European regions. Otherwise, it would be challenging to reconcile this new hypothesis with the geographical progression of evolving genotypes within the A.Br.WNA clade. This progression runs from northern North America (Canada) along the North American prairies towards the south [5]. French settlers likely migrated from the northwest into the new continent [6]. Spanish conquistadors and colonists arrived through the Caribbean to the mainland but not to Canada, e.g., the Spanish Hernando de Soto was the first European adventuring through the southeast of North America into what is nowadays known as the United States. He was also the first to cross the Mississippi River, by 1540 [20,22].

Thus, there are still gaps in our understanding of events leading to trans-continental pathogen displacement as well as the temporal and spatial resolution of *B. anthracis* evolution past the A.Br.147 SNP divergence. This is largely due to the lack of genomic information for *B. anthracis* imported into regions of the Colonial Spanish Empire after 1492 and of motherland Spain itself. Especially, the Mexico of today could provide clues on missing phylogenetic links. *B. anthracis* is present in Mexico (and many other Latin American countries but not on most of the Caribbean Islands) [1,24]. Unfortunately, very little information is publicly available regarding *B. anthracis* genotypes present in Mexico (and in other southern North American countries). The little that is available of genome sequences in databases does not fall into the A.Br.011/009 clade except for a strain from Mexico that is A.Br.WNA [2,7]. Information from southern Latin American countries certainly could substantiate phylogenetic links; however, previously reported *B. anthracis* genotypes from South America mainly belong to the A.Br.003/004 clade [7] (now A.Br.054 or V770 [2]).

This only loose correlation (in most cases) of a genotype with a geographic region does complicate the *B. anthracis* attribution of strains when genomic analysis is conducted. The situation is similar in France and Spain. Besides A.Br.009/11 isolates, France also comprises *B. anthracis* from such clades as diverse as A.Br.001/002 (Sterne) and B.Br.004 (CNEVA) [6]. For Spain, a previously reported *B. anthracis* genome grouped with the A.Br.007 (Vollum) clade [2]. No further information on the geographic location and date of isolation as well as its host is available. A little better is the situation for strains 319/02 and 342/02, which have been previously genotyped [3,25]. These human clinical isolates likely originated in central Spain (Guadalajara and Zaragoza, respectively) in 2002. In our previous work, strain 319/02 was thought to be unique among the Spanish isolates because it featured the longest branch of any of the analyzed chromosomes with 432 presumable autapomorphic SNP positions [3]. It was hypothesized that such a high number of autapomorphic sites in this and other remarkable isolates renders them “mutator strains” with high intrinsic mutation rates [3,26]. However, we have now re-sequenced the genomes of strains 319/02 and 342/02. With the higher sequence depth now achieved, the number of autapomorphic sites diminished to 19 (previously 432) in strain 319/02 and to 32 (previously 127) in strain 342/02. These numbers match those of most other strains in the A.Br.011/009 phylogeny much better than before.

The diversity of most of the recent *B. anthracis* outbreak isolates from Spain (the A.Br.11/ESPc branch) is very low. Only 31 SNPs were identified within this group with a maximum distance of 12 SNPs between any chromosomes (Figure 2B). This shallow branching is somewhat in contrast to the relatively large area from where the respective strains were isolated in 2021 (approximately 4000 km^2^). Within the A.Br.11/ESPc clade, the length of the individual distances between isolates varied from one to eight SNPs. Numbers above five are considered quite high for single *B. anthracis* outbreaks. In previous studies, this threshold of five (SNPs or alleles) was set to define isolates from a connected anthrax outbreak event. In one study, the authors investigated two different outbreaks. In both instances, strains collected from these outbreaks differed by only up to five genomic alleles [27]. Similarly, analysis of chromosomal SNPs from another outbreak also confirmed a distance of five alleles (i.e., comparable to SNP distances) [18]. Thus, the 2021 outbreak in Extremadura might not be a single but rather two spatio-temporal overlapping outbreaks. Alternatively, this data could be interpreted speculatively in that at least at the farms near Acedera, the disease foci were very active with a high number of bacterial generations introducing additional mutations above the preset threshold. Oddly, identical *B. anthracis* genotypes were found at farms that were up to 83 km apart. Likewise, four farms yielded different genotypes with up to eight SNP differences among these strains. The cause of this spatial distribution remains unresolved at the time being. Anthropogenic activities (such as animal or animal product trafficking) could be responsible, or the cause could be natural as, e.g., birds of prey might feed on anthrax-diseased prey and spread the disease [28]. Indeed, the movement of animals or their products was an initial hypothesis of pathogen spread. However, when an anthrax outbreak is declared, the restriction of these movements is the main measure adopted by the authorities and carcasses are transported in sealed trucks for disposal at authorized waste plants. The role of insects, such as horseflies, was strongly considered. In this sense, several insect traps were analyzed. Although strain isolation could not be achieved, *B. anthracis* DNA could be detected by PCR in one of them (farm 21 in Villanueva de la Serena).

Of note, both outbreaks (Ciudad Real and Extremadura) are located in the hydrographic basin of the Guadiana River. A network of rivers running very close to the affected farms (Guadiana River and its tributaries Zújar River, Ruecas River, Gargáligas River, Ortiga River, Búrdalo River, Guadalmez River) might connect the Extremadura outbreak event. Two findings suggest a possible contribution related to the hydrography of the terrain aiding in the spread of the pathogen. First, different genotypes were found on the same farm (farm 5: 2 genotypes, farm 13: 3 genotypes, farm 17: 2 genotypes and farm 18: 2 genotypes) and in different geographical locations (Navalvillar de Pela, Acedera, Logrosán and Garbayuela). Second, the same genotype was found to be distributed across several towns (genotype 21/903-1-like in Talarrubias, Navalvillar de Pela, Mérida and Garbayuela; genotype 21/926-5-like in Garbayuela, Navalvillar de Pela and Villanueva de la Serena).

Apart from the outbreak investigation, the important detail in the phylogeny of the A.Br.11/ESPc lineage is the quite short branch lengths to the nodes eventually leading to West African strains and to the node at the base of A.Br.WNA (31 SNPs each; 29+2). This makes isolates from clade A.Br.11/ESPc the closest living relatives of A.Br.WNA strains. The observed very different numbers of autapomorphic SNPs of clade A.Br.WNA vs. A.Br.11/ESPc also strengthen the hypothesis of accelerated evolution once *B. anthracis* had reached new territories, i.e., the anthrax-naïve animal population in America [6,7,29].

## 5. Conclusions

The A.Br.WNA clade of *B. anthracis* is thought to be exclusively distributed across (North) America. The colonization of this continent by this clade appears to have commenced through an introduction from Europa rather than from Asia and occurred only a couple of centuries ago. Previous genomic data suggested that the origin region of these founder individuals is southwestern Europe, possibly France, Italy or Spain. From there, the anthrax pathogen immigrated along with human activities to the New World. The hitherto closest extant relatives of the American A.Br.WNA descendants have now been isolated from recent anthrax outbreaks in Spain. In order to substantiate the hypothesis that Spain was a likely origin of this founder population of American *B. anthracis*, more isolates from Spain (and possibly from bordering French departments as well as southern Italy) are needed. Finally, the usefulness of the DMAA PCR SNP-typing approach has been supported in this study as it can interrogate for clade-specific SNPs previously identified by whole genome SNP-discovery in additional strains.

## Figures and Tables

**Table 1:** *B. anthracis* strains isolated from outbreaks in Spain in 2021 ^1^.

Strain Designation ^2^	Region of Isolation (Province)	Farm Code	Host
**21/868**	Ciudad Real (Ciudad Real)	A	Cattle
**21/874**	Navalvillar de Pela (Badajoz)	1	Horse
**21/878**	Navalvillar de Pela (Badajoz)	3	Horse
**21/878-2**	Casas de Don Pedro (Badajoz)	2	Cattle
21/890-8	Navalvillar de Pela (Badajoz)	5	Cattle
21/890-9	Navalvillar de Pela (Badajoz)	4	Cattle
21/891-2	Navalvillar de Pela (Badajoz)	7	Horse
**21/903-1**	Talarrubias (Badajoz)	9	Cattle
21/903-6	Navalvillar de Pela (Badajoz)	4	Cattle
21/905-13	Navalvillar de Pela (Badajoz)	5	Pig
21/918-3	Talarrubias (Badajoz)	9	Cattle
**21/920-1**	Logrosán (Cáceres)	12	Cattle
**21/920-14**	Navalvillar de Pela (Badajoz)	5	Pig
**21/924-2**	Acedera (Badajoz)	13	Cattle
**21/925-5**	Acedera (Badajoz)	13	Cattle
**21/926-3**	Logrosan (Cáceres)	17	Cattle
**21/926-5**	Garbayuela (Badajoz)	18	Cattle
21/926-9	Navalvillar de Pela (Badajoz)	15	Cattle
21/926-22	Mérida (Badajoz)	14	Cattle
**21/933-1**	Logrosán (Cáceres)	17	Cattle
**21/940-6**	Acedera (Badajoz)	13	Cattle
**21/940-10**	Logrosán (Cáceres)	8	Cattle
21/940-19	Navalvillar de Pela (Badajoz)	n.d.	Wild boar
21/955-9	Garbayuela (Badajoz)	18	Cattle
**21/984-4**	Logrosán (Cáceres)	19	Cattle
**21/997-5**	Acedera (Badajoz)	20	Horse
21/1088-3	Villanueva de la Serena (Badajoz)	21	Cattle
**21/1088-10**	Don Benito (Badajoz)	22	Cattle
**21/1198-6**	Madrigalejo (Cáceres)	23	Cattle

^1^ details in Table S1. ^2^ Chromosomes of strains set in bold type were used for further genomic analysis. n.d.: not determined because the disease affected a wild animal (not from a farm).

## Data Availability

The genome sequence data presented in this study are available from the NCBI database under the BioProject ID: PRJNA930337. These and the accession numbers of publicly available genome sequences analyzed are listed in the Supplementary Material (Table S2) of this manuscript.

## Supplementary Materials

The following supporting information can be downloaded at: https://www.mdpi.com/article/10.3390/microorganisms11040889/s1, Table S1: anthrax outbreaks in Spain 2021; Table S2: genome sequences—accession numbers; Table S3: SNP matrix and Table S4: primers.
